# Chinese expert consensus on diagnosis and treatment of coagulation dysfunction in COVID-19

**DOI:** 10.1186/s40779-020-00247-7

**Published:** 2020-04-20

**Authors:** Jing-Chun Song, Gang Wang, Wei Zhang, Yang Zhang, Wei-Qin Li, Zhou Zhou, People’s Liberation Army Professional Committee of Critical Care Medicine, Chinese Society on Thrombosis and Haemostasis

**Affiliations:** 1 Department of Critical Care Medicine, the 908th Hospital of Joint Logistics Support Forces of Chinese PLA, Nanchang, 330002 China; 2 Department of Critical Care Medicine, the Second Affiliated Hospital of Xi’an Jiaotong University, Xi’an, 710001 China; 3 Department of Emergency Medicine, the 900th Hospital of Joint Logistics Support Forces of Chinese PLA, Fuzhou, 350000 China; 4 Department of Laboratory Medicine, Fuwai Hospital, Chinese Academy of Medical Sciences, Peking Union Medical College, Beijing, 100037 China; 5 Department of Critical Care Medicine, General Hospital of Eastern Theater Command of Chinese PLA, Nanjing, 210002 China

**Keywords:** COVID-19, Severe, Coagulation dysfunction, Diagnosis, Treatment

## Abstract

Since December 2019, a novel type of coronavirus disease (COVID-19) in Wuhan led to an outbreak throughout China and the rest of the world. To date, there have been more than 1,260,000 COVID-19 patients, with a mortality rate of approximately 5.44%. Studies have shown that coagulation dysfunction is a major cause of death in patients with severe COVID-19. Therefore, the People’s Liberation Army Professional Committee of Critical Care Medicine and Chinese Society on Thrombosis and Hemostasis grouped experts from the frontline of the Wuhan epidemic to come together and develop an expert consensus on diagnosis and treatment of coagulation dysfunction associated with a severe COVID-19 infection. This consensus includes an overview of COVID-19-related coagulation dysfunction, tests for coagulation, anticoagulation therapy, replacement therapy, supportive therapy and prevention. The consensus produced 18 recommendations which are being used to guide clinical work.

## Background

Since December 2019, an outbreak of coronavirus disease 2019 (COVID-19) in Wuhan, China, has spread throughout the world [1]. On February 11, 2020, the Coronavirus Study Group (CSG) of the International Committee on Taxonomy of Viruses (ICTV) officially named the novel coronavirus that caused this COVID-19 epidemic as ‘severe acute respiratory syndrome coronavirus 2 (SARS-CoV-2)’ [2]. As of April 5, there has been 1,267,174 cases of COVID-19 worldwide, of which 68,931 patients have died, with a mortality rate of approximately 5.44% [3]. Coagulation dysfunction is one of the major causes for death in patients with severe COVID-19 [4]. Previous studies have shown that 71% of the patients who died have met the criteria set by the International Society on Thrombosis and Haemostasis (ISTH) for the diagnosis of disseminated intravascular coagulation (DIC) [5]. Therefore, the People’s Liberation Army Professional Committee of Critical Care Medicine and Chinese Society on Thrombosis and Hemostasis gathered experts from the epidemic frontline of Wuhan to establish a committee to reach a consensus. This consensus includes an overview of COVID-19-related coagulation dysfunction, coagulation tests, anticoagulation therapy, replacement therapy, supportive therapy and prevention. There are 18 recommendations which are being used to provide guidance for relevant clinical work.

## Overview

### Recommendation 1: Severe COVID-19-related coagulation dysfunction has a pathological basis

Novel coronaviruses belonging to the β-type coronavirus and SARS viruses both utilize the S protein to bind to an angiotensin converting enzyme (ACE) 2 protein on the cell surface membrane, in order to enter into human cells [6]. However, the S protein trimeric structure of the novel coronavirus is more likely to bind to the ACE2 protein on the cell surface, resulting in the novel coronavirus S protein having a 10 to 20 times higher affinity to ACE2 when compared to the SARS virus [7]. ACE2, the main target of the novel coronavirus, is mainly distributed in the heart, lungs, kidneys, testes, and digestive tract [8]. Therefore, the pathological results showed that SARS-CoV were detected in alveolar type II epithelial cells, monocytes, digestive tract epithelial cells, distal renal tubulesepithelial cells, skin sweat gland cells, parathyroid and pituitary eosinophils, adrenal cortex cells, gastric parietal cells, pancreatic acinar cells and tracheal serous gland cells. Different from SARS-CoV, SARS-CoV-2 were mainly detected in alveolar type II epithelial cells and pulmonary macrophages, and partly in hilar lymph nodes, spleens and testes [9]. Coronavirus has an extensive tissue distribution, causing a high number of proinflammatory cytokines to be released, promoting a systemic inflammatory response syndrome (SIRS), accelerating cell death in the lungs, livers, heart, kidneys and the adrenal parenchymal organs, which can ultimately lead to multiple organ dysfunction syndrome (MODS) [10]. As inflammatory reactions occur in the all organs of the body, the microvascular system is damaged, leading to abnormal activation of the coagulation system, which pathologically manifests as generalised small vessel vasculitis and extensive microthrombosis [9, 11]. Previous study has shown that an abnormal urokinase pathway can cause a SARS-CoV -induced lung injury [12], and suggested that the interaction between novel coronavirus, coagulation and fibrinolytic systems has a complicated molecular mechanism, which requires further study.

## Evaluation of blood coagulation function

### Recommendation 2: The past medical history should be fully understood and the patients’ coagulation function should be accurately evaluated in severe SOVID-19 patients.

The epidemiological analysis of 72,314 cases of COVID-19 in China conducted by Chinese Center for Disease Control demonstrated that most of the deaths related to COVID-19 were aged more than 60 years with underlying conditions such as hypertension, coronary heart disease, diabetes, etc. [13]. Current consensus of COVID-19 included patients aged 65 years or those complicated with severe chronic underlying diseases (such as hypertension, diabetes, coronary heart disease, malignancy, structural lung disease, pulmonary heart disease and immunosuppression) as critically ill individuals [14]. The COVID-19 patients who having serious underlying conditions might be at high risk of coagulation dysfunction. Therefore, obtaining the details of severe COVID-19 patients is necessary (Table 1).

**Table 1:** Underlying conditions that may cause coagulation dysfunction

Cause	Disease type
Factors that increase the risk of bleeding	Hereditary coagulation factor deficiency (such as hemophilia), fibrinogen reduction, platelet deficiency, etc.
	Acquired clotting factor reduction, hyperfibrinolysis, thrombocytopenia, etc.
	History of taking anticoagulant or antiplatelet drugs
	Diseases such as bronchiectasis, peptic ulcer, liver cirrhosis and hemorrhoids
	24 h after severe trauma or surgery
Factors that cause thromboembolism	Congenital deficiency of anticoagulants (eg, antithrombin (AT) deficiency, protein C/S deficiency)
	Diabetes, systemic lupus erythematosus, antiphospholipid syndrome, systemic vasculitis and other underlying diseases which have vascular endothelial cells damage
	History of thrombotic diseases such as venous thrombosis, cerebral thrombosis, arterial embolism, myocardial infarction
	A state of shock
	Bed-ridden
	Surgery
	Complications with acute infections

### Recommendation 3: Routine coagulation tests are recommended to screen coagulation function for severe COVID-19 patients

Routine coagulation tests include: 1) Evaculation of exogenous coagulation system status assessment: prothrombin time (PT) or International Standardized Ratio (INR). Prolonged PT or INR indicates an abnormal quantity or quality of coagulation factors in the exogenous coagulation system, or the presence of anticoagulants in the blood. 2) Evaculation of endogenous coagulation system status assessment: activated partial thromboplastin time (APTT). A prolonged APTT indicates an abnormal quantity or quality of coagulation factors in the endogenous coagulation system, or the presence of anticoagulant substances in the blood. A shortened APTT indicates the hypercoagulable status. APTT can also be used to determine heparin dosage. 3) Evaluation of common coagulation pathways: thrombin time (TT) and fibrinogen. Prolonged TT and normal fibrinogen level indicates the presence of anticoagulant substances in the blood. Prolonged TT and decreased fibrinogen level suggest hypofibrinogenemia. 4) Evaluation of fibrinolytic system: D-dimers (DD) and fibrin degradation products (FDP). Increased DD and FDP suggests the fibrinolytic system activity. 5) Platelet (PLT) count: if the number of PLT decreases, the risk of bleeding increases; if the number of PLT increases, PLT are prone to adhesion, aggregation and release reactions, increasing the risk of platelet thrombosis.

The retrospective analysis of 99 COVID-19 patients conducted by Wuhan Jinyintan Hospital showed that 36% of the patients showed an elevated DD, 16% showed a reduced APTT, 6% showed an extended APTT, 30% showed a shortened PT and 30% showed an extended PT [15]. Professor Tang, an expert on the committee, conducted a retrospective analysis of the routine coagulation parameters of 183 patients with COVID-19, and found that plasma FDP and DD in non-survivors were significantly higher than those in survivors; PT and APTT were significantly prolonged [5]. A retrospective analysis of 138 COVID-19 patients by Professor Peng also confirmed that D-dimers increased after admission [16]. Previous studies evidenced that elevated DD is an independent risk factor for acute respiratory distress syndrome (ARDS) and mortality in COVID-19 patients [4].

### Recommendation 4: ISTH/CDCC DIC scores are recommended to diagnose COVID-19-related coagulation dysfunction

DIC is a secondary syndrome characterized by intravascular coagulation due to local damage. It is a manifestation of coagulation failure and an intermediate link in the development of multiple organ failure (MOF). Professor Tang’s retrospective analysis of 183 patients with COVID-19 showed that DIC occurred in 74.1% non-survivors and 0.6% survivors [5]. Presently, DIC is based mainly on the diagnostic criteria of the Scientific and Standardization Committee (SSC) of the ISTH. The parameters of DIC diagnostic scoring system included PT, PLT, fibrinogen and DD. A score of ≥5 can be diagnosed as overt DIC and < 5 is diagnosed as non-overt DIC, which would need to be evaluated daily [17] (Table 2). In 2017, the Thrombosis and Hemostasis Group of the Chinese Medical Association Hematology Branch recommended the Chinese DIC scoring system (CDSS), which added the primary disease and revised the diagnostic thresholds [18, 19].

**Table 2:** Diagnostic criteria for DIC

Index	Score	ISTH	CDSS
PLT (×10^9^/L)	0	> 100	> 100
	1	≤100	80–100
			24 h decrease ≥50%
	2	≤ 50	< 80
PT/APTT (s)	0	PT extension < 3	PT extension < 3 and APTT extension < 10
	1	PT extension ≥3 and < 6	PT extension ≥3 or APTT extension ≥10
	2	PT extension ≥6	PT extension ≥6
Fibrin-related indicators, (μg/mL)	0	DD < 2.5	
	1	–	< 5
	2	Moderate increase, 2.5 ≤ DD < 5.0	5 ≤ DD < 9
	3	Severe increase, DD ≥ 5.0	≥9
Fibrinogen (g/L)	0	≥1.0	≥1.0
	1	< 1.0	< 1.0
Underlying disorder known to be associated with DIC	0	necessary	
	2		presence
Clinical manifestation	1		abnormal bleeding;unexplained organ failure; shock or microcirculatory disorder (meet anyone 1 point)
Total score		≥ 5	≥ 7

### Recommendation 5: Viscoelastic tests are recommended for severe COVID-19 patients with coagulation dysfuntion

The viscoelastic tests use whole blood to reflect the coagulation status of patients with existing coagulation dysfunction fully and accurately [20]. Therefore, it is recommended that viscoelastic tests are used to evaluate the coagulation function of severe COVID-19 patients. Viscoelastic coagulation tests mainly include a thromboelastograph (TEG) and a coagulation and platelet function analyzer (Sonoclot^@^ or Centuryclot^@^). The main indicators of TEG include R-time for coagulation factor activity, α angle and k-time for fibrinogen function, MA for platelet function, and LY30% for fibrinolytic function. The main indices of Sonoclot or Centuryclot include: ACT indicates coagulation factor function, CR indicates fibrinogen function, and PF indicates platelet function. TEG and Sonoclot/Centuryclot can accurately assess the overall coagulation status of patients. TEG and Sonoclot/Centuryclot show a good correlation with the assessment of coagulation factors, fibrinogen and PLT of critically ill patients. Moreover, Sonoclot/Centuryclot show more sensitivity in monitoring coagulation factors and platelet function. TEG has great advantages in monitoring fibrinolytic function [21].

## Anticoagulation therapy

### Recommendation 6: Anticoagulant therapy can be utilized without anticoagulant contraindications in severe COVID-19 patients with thrombosis

Severe COVID-19 patients with a thromboembolic disease are more likely to be hypercoagulable state. Routine coagulation assays may show shortened PT or APTT in these patients [15], and viscoelastic tests may show coagulation factors and platelet hyperfunction [22]. The significant increasing in DD also indicates the possibility of thrombosis, represented as thrombosis events such as deep vein thrombosis, pulmonary embolism, myocardial infarction and cerebral infarction. Anticoagulant therapy is recommended without anticoagulant contraindications. Parenteral anticoagulant drugs are preferred, with choice of drug and dosage depending on the location and severity of the embolism [23].

### Recommendation 7: In severe COVID-19 patients with coagulation dysfunction, anticoagulant therapy using unfractionated heparin/low-molecular-weight heparin are recommended to reduce the depletion of coagulation substrates

Severe COVID-19 patients can activate the coagulation pathway because of inflammatory cytokine storms, which can cause excessive consumption of coagulation factors and platelets [16]. Moreover, excessive depletion of coagulation substrates causes coagulation dysfunction leading to the development of DIC and even MOF. In order to reduce the coagulation substrate depletion, some experts suggested general COVID-19 patients should be given 1 to 2 doses of low molecular heparin daily, until the patient’s DD level returns to normal. Once FDP ≥ 10 g/mL and/or DD ≥ 5 μg/mL, unfractionated heparin (3–15 IU/kg per hour) should be used. The coagulation function and platelets must be re-evaluated 4 hours after the initial heparin treatment [24]. Some researchers have conducted a retrospective analysis of patients with COVID-19 and discovered that using low-molecular-weight heparin (LMWH) anticoagulation for patients with a DD value greater than 6 timescan significantly reduce mortality, compared with those who have not received low-molecular-weight heparin treatment (not published). However, this treatment plan currently requires more evidence.

For severe COVID-19 patients with coagulation dysfunction, unfractionated heparin/LMWH are recommended for anticoagulant therapy [25]. Unfractionated heparin has the advantages of a short half-life, a convenient monitoring process and the fact that it can be neutralized with protamine. Therefore, it is recommended that unfractionated heparin should be the first choice. It is also recommended that unfractionated heparin should be administered intravenously. The dosage should be determined according to the coagulation function and organ function. Those with severe coagulation dysfunction can start with a low dose [1 U/(kg∙h)] and use laboratory indicators to guide titration. LMWH is not easy to adjust or monitor due to its long half-life, therefore it is recommended only to be used in mild to moderate coagulation dysfunction. The initial dose is generally recommended to be given as 1 mg/kg q12h intravenously or subcutaneously, and the dosage should be monitored according to anti-Xa activity with the target range of 0.6~1.0 IU/mL. LMWH is metabolized by the kidneys, and therefore, patients with kidney dysfunction need to be monitored. The ATIII activity of severe COVID-19 patients should be maintained at > 80%, otherwise it may affect the effect of the anticoagulant. If ATIII activity is reduced, it can be increased by supplementing fresh frozen plasma.

### Recommendation 8: In severe COVID-19 patients with coagulation dysfuntion, a TEG heparinase comparison test is recommended to monitor unfractionated heparin dosage

Most COVID-19 patients with coagulation dysfunction receive heparin. Routine coagulation and viscoelastic tests reflect the combined results from coagulation dysfunction and heparin administration. Therefore, it is difficult to make an accurate assessment of real coagulation function clinically. The TEG heparinase comparative test is recommended to evaluate the coagulation function of severe COVID-19 patients undergoing heparin anticoagulation therapy and to monitor unfractionated heparin dosage [26]. The TEG heparinase comparison test requires the control tube and the heparinase tube to be tested at the same time. The control test represents the combined effects of the patient’s own coagulation dysfunction and the effect of heparin. The heparinase tube test can accurately reflect the patient’s own coagulation status by breaking down heparin in the blood, and thus can be used to titrate the heparin dosage. It is generally recommended that the ratio of the R time in the control tube to the R time in the heparinase tube is approximately 2 [27].

### Recommendation 9: For severe COVID-19 patients with coagulation dysfunction requiring continuous renal replacement therapy, unfractionated heparin/LMWH for systemic anticoagulation or no anticoagulation is recommended

In continuous renal replacement therapy (CRRT), anticoagulation methods can be divided into systemic anticoagulation, local anticoagulation and no anticoagulation. Systemic anticoagulation is a method that reduces the blood coagulation by using anticoagulant drugs intravenously. Local anticoagulation is a method that only reduces the blood coagulation in the extracorporeal circuit without affecting the blood coagulation in the body. If circuit anticoagulation is the goal, local anticoagulation or systemic anticoagulation can be used. If patient anticoagulation is the goal, systemic anticoagulation is recommended to alter the coagulation status for treatment of the primary disease. To avoid an increased risk of bleeding, the dose of anticoagulant drugs needs to be adjusted according to the patient’s coagulation status. The recommended dose of unfractionated heparin is shown in Table 3. The blood status of the patient needs to be monitored within 6 h of anticoagulant administration, and then monitored regularly depending on the severity of their condition. A TEG heparinase comparison test is recommended to titrate unfractionated heparin dosage with the R/Rh ratio as 2 [27]. It is a good method to titrate the dose of unfractionated heparin according to the change of APTT, TEG R-time or Sonoclot/Centuryclot ACT from the previous base value. Protamine can be considered in the case of a severe heparin overdose.

**Table 3:** Different heparin anticoagulant doses determined by the risk of bleeding

Loading dose (IU/kg)	Maintenance dose IU/(kg∙h)	INR	APTT (s)	PLT (×10^**9**^/L)
30 ~ 50	10	≤ 1.5	≤ 40	≥ 150
20–40	5	> 1.5 but ≤2.5	> 40 but ≤60	< 150 but ≥70
No	Citrate anticoagulation or no anticoagulation	> 2.5	> 60	< 70

The use of low-molecular-weight heparin can be repeated with a bolus or by continuous pumping. It is recommended that a repeated bolus should initially be an intravenous dose of 60 ~ 80 IU/kg, taken 20 ~ 30 min before treatment, with an additional intravenous dose of 30 ~ 40 IU/kg taken every 4 ~ 6 hours. The additional doses should be gradually reduced as the treatment period goes on. The activity of plasma anti-Xa should be monitored and maintained at 0.3 IU/ml, and the LMWH dose should be adjusted based on the results. The LMWH rate of continuous pumping also needs to be adjusted based on the measurements of the plasma anti-Xa activity.

### Recommendation 10: Topical citrate anticoagulation is recommended for severe COVID-19 patients with active bleeding who require CRRT

Severe COVID-19 patients with active bleeding who require CRRT should be treated with local citrate anticoagulation to avoid aggravating coagulation disorders. 4% trisodium citrate solution should be continuously administered before the filter at a dose of 1.2 to 1.5 times the blood flow rate (ml/min). The citric acid concentration before the filter should reach 3 to 4 mmol/L, the free calcium after the filter should be maintained at 0.25 ~ 0.35 mmol/L and the venous free calcium should be at 1.1 ~ 1.35 mmol/L. The citric acid dose should be adjusted depending on the concentration of the ionized calcium concentration after the filter, and the calcium chloride or calcium gluconate solution dose should be adjusted depending on the serum ionized calcium concentration in the body [28].

### Recommendation 11: For severe COVID-19 patients with coagulation dysfunction requiring external membrane oxygenation therapy, unfractionated heparin is the preferred anticoagulant and there should be more monitoring of their coagulation status

Severe COVID-19 cases are mainly defined by the damage done to the lungs. Extracorporeal Membrane Oxygenation (ECMO) is an important treatment method for critically ill COVID-19 patients. Heparin is the most commonly used anticoagulant in ECMO adjuvants, but it also increases the risk of bleeding [29]. Bleeding in severe COVID-19 patients with coagulation dysfunction is more common at surgical incision or ECMO intubation sites, in the nose and in airways. Gastrointestinal bleeding can also occur, the most serious of which is intracranial bleeding. Therefore, in severe COVID-19 patients with coagulation dysfunction, there needs to be increased monitoring of the coagulation function during ECMO adjuvants. Monitoring methods commonly involve activated clotting time (ACT) and APTT. ACT should be maintained at 180 ~ 220 s and/or ACT and APTT should be maintained at 1.5 times the normal upper limit. The TEG heparinase comparison test is also applicable to ECMO anticoagulation monitoring [30]. For detailed methods refer to ‘Recommendation 8’. If the anti-Xa activity is used to monitor the heparin dose, it is recommended that Xa activity be maintained at 0.3 ~0.7 IU/ml. During ECMO plus CRRT treatment, anticoagulation strategy and monitoring are similar to receiving ECMO only.

### Recommendation 12: If heparin-induced thrombocytopenia occurs in severe COVID-19 patients, the anticoagulant argatroban/bivalirudin is recommended

Heparin-induced thrombocytopenia (HIT) is a disease caused by platelet-activated antibodies during treatment with heparin. It mainly manifests as thrombocytopenia, as well as venous and arterial thrombosis, and even death. Literature review shows that the incidence of HIT is approximately 0.1% to 5.0%, and the incidence is higher with unfractionated heparin use than with LMWH use [31]. HIT is mainly caused by platelet factor 4 (PF4)-heparin complex, which stimulates immune cells to produce PF4-heparin complex antibodies (i.e., HIT antibodies), which in turn leads to persistent platelet activation, activation of the coagulation pathway, platelet thrombosis, and fibrin thrombus. If a severe case of COVID-19 leads to a decrease in the platelet count at > 50% of the basal value and/or shows signs of arteriovenous thrombosis after an administration of heparin, the 4 T’s score or HIT antibody can be used to diagnose HIT. The 4T's score is a validated clinical prediction tool for HIT, and it is composed of elements related to the degree of thrombocytopenia, the time to thrombocytopenia from heparin product exposure, the associated thrombotic complications and the concurrent presence of other potential etiologies of thrombocytopenia. A score ≤ 3 is classified as low likelihood of HIT, 4 ~ 5 is moderate, and 6~8 is highly possible. The diagnosis can be essentially confirmed if the 4 T’s score is ≥4 and the HIT IgG-specific antibody is positive [32].

For patients with a high likelihood of or confirmed HIT, the use of heparin should be discontinued and they should be switched to non-heparin anticoagulants such as argatroban or bivalirudin [33]. 1) Argatroban: Patients who are allergic to heparin or HIT can choose argatroban anticoagulation. Argatroban is a direct thrombin inhibitor that is metabolized in the liver and can cause a significant increase in TT. The recommended dose starts with an intravenous argatroban infusion [0.2–0.5 μg/(kg∙min)], and the dose should be adjusted based on the dynamic change of APTT, TEG R-time, Sonoclot/Centuryclot ACT value. In patients with moderate liver dysfunction and heart failure, argatroban should be initially administered by an intravenous infusion at 0.5–1.2 μg/(kg∙min). In the case of multiple organ dysfunction, argatroban can be administered by intravenous infusion at 0.2–0.5 μg/(kg∙min). 2) Bivalirudin: The effective anticoagulant component is a hirudin derivative fragment which directly and specifically inhibits thrombin activity. The effect is short-lived (half-life 25–30 min) and reversible. It can be applied to patients with HIT or suspected HIT. The starting dose is 0.05 mg/kg/h and this should be adjusted depending on the APTT, TEG R-time or dynamic changes in Sonoclot/Centuryclot ACT values.

## Replacement treatment

### Recommendation 13: Goal-directed replacement therapy is recommended for severe COVID-19 patients with coagulation dysfunction

Goal-directed replacement therapy is recommended for severe COVID-19 patients with coagulation dysfunction, which involves routine coagulation indicators, TEG parameters and Sonoclot/Centuryclot indices [34].

When PT or APTT is prolonged by > 1.5 times, or TEG R-time is > 10 min, or Sonoclot/Centuryclot ACT is > 240 s (anticoagulated whole blood), fresh frozen plasma should be infused at 15 to 30 ml/kg as soon as possible. After the infusion, coagulation indicators can be dynamically monitored to determine the additional infusion dose. If an excessive fluid load is present in the patient, coagulation factors can be supplemented in combination with prothrombin complex concentrate [35]. If fibrinogen < 1.5 g/L, or TEG functional fibrinogen level test indicator FF_ma_ is < 10 mm or Sonoclot/Centuryclot CR is < 10, cryoprecipitated fibrinogen (10 ml/kg) or human fibrinogen (30–50 mg/kg) can be infused. Coagulation indicators can be dynamically monitored to maintained the plasma fibrinogen at least 1.5 g/L by the additional infusion [36].

For severe COVID-19 patients who are not bleeding, platelet transfusion is recommended if the platelet count is  < 20 × 10^9^/L; for severe COVID-19 patients who intend to undergo a lumbar puncture or present with active bleeding, platelet transfusion is recommended if the platelet count is < 50 × 10^9^/L; for severe COVID-19 patients undergoing ECMO, platelet transfusion is recommended if the platelet count is < 80 × 10^9^/L [30, 37]. Previous studies have shown that the use of platelet count as an indication for platelet transfusions has not been shown to improve the outcomes of critically ill patients [38]. However, the use of platelet function indicators to guide platelet transfusions in critically ill patients with thrombocytopenia may be beneficial for improving the outcome of these patients [39]. Studies have shown that platelet transfusion is recommend if TEG MA < 43 mm [40] or Sonoclot/Centuryclot PF < 1 [41]. For patients receiving antiplatelet therapy, platelet transfusion is recommended in cases of persistent bleeding with platelet dysfunction and even thrombocytopenia [42]. The platelet transfusion amount should be individualized, taking into account the patient’s weight, spleen function, and other depletion factors. The dose is generally 1 unit of apheresis platelets or an equivalent dose of platelet concentrate per dosing; ≥ 2 units of apheresis platelets may be transfused in the case of severe life-threatening bleeding. After the infusion, the dose should be evaluated and adjusted according to the effect. The goal is to transfuse the minimum dose required to maintain the platelet count target. One unit of platelet transfusion can theoretically increase platelets from 4 × 10^9^/L to 8 × 10^9^/L (70 kg) in adults.

### Recommendation 14: If bleeding is not effectively stopped after active replacement therapy in severe COVID-19 patients, recombinant factor VII is recommended

If hemorrhage is not effectively stopped by active replacement therapy and a hypocoagulation state is still indicated by coagulation monitoring, recombinant factor VII (rVII) can be used. In order to more effectively stop the bleeding, the following conditions must be met when using rVII: 1) Acidosis, hypothermia, and hypocalcemia should be corrected; 2) Hematocrit < 24%, platelet count < 50 × 10^9^/L, fibrinogen< 1.5 g/L. The initial dose of rVII should be 100–200 μg/kg. Depending on the state of the bleeding, rVII 100 μg/kg can be given at intervals of 2 hours. The drug can be discontinued based on the amount of bleeding and the coagulation test results [43].

## Supportive treatment

### Recommendation 15: In severe COVID-19 patients with coagulation dysfunction experiencing liver failure, plasma exchange is recommended

Severe COVID-19 patients with hepatic encephalopathy of grade II or higher and the following manifestations can be diagnosed with acute liver failure [44]: 1) obvious digestive symptoms such as anorexia, abdominal distension, nausea or vomiting; 2) progressive jaundice with serum total bilirubin ≥10 times the normal upper limit in short time or a daily increase ≥17 μmol/L; 3) prothrombin activity ≤40% or an INR ≥1.5 excluding the other causes; 4) progressive liver shrinkage.

In severe COVID-19 patients with coagulation dysfunction experiencing liver failure, artificial liver support system (ALSS) is recommended. Plasma exchange is a widely used and effective treatment in ALSS. Therapeutic PE is a process by which plasma is separated from blood cells by centrifugation or membrane separation, discarded, and then replaced by fresh-frozen plasma. Large-molecular-weight substances including autoantibodies, immune complexes, cholesterol, bilirubin, drugs and poisons, are removed from the plasma by PE [45]. At the same time, coagulation factors are supplemented to improve the coagulation status of the critically ill patients. Previous studies have confirmed that in septic shock patients using large doses of vasoactive drugs to maintain blood pressure, early plasma exchange can decrease the dosage of vasoactive drugs, remove inflammatory cytokines, reduce capillary leakage and platelet consumption [46, 47].

If plasma is sufficient, PE should be performed in combination with plasma adsorption, perfusion and/or hemofiltration [plasma exchange volume (L) = body mass (kg) (1/13) (1-hematocrit/100)]. If plasma is deficient, PE should be performed with more than 2000 ml plasma. If plasma is unavailable or less than 2000 ml, plasma adsorption, perfusion, hemofiltration and their combination therapy are recommended [48]. Viscoelastic tests are recommended to evaluate coagulation function and monitor anticoagulation therapy in critically ill patients with liver failure [49].

## Prevention

### Recommendation 16: For severe COVID-19 patients with coagulation dysfunction, heparin flush during vascular access placement should be avoided

Severe COVID-19 patients are often inserted central venous catheters (CVCs) for treatment. CVCs can be used for monitoring hemodynamic status, administration of parental nutrition, drugs and blood products, and performing of hemodialysis. Sometimes invasive intra-arterial blood pressure monitoring should be performed for hemodynamically unstable patients. To maintain the potency of vasular access devices, repeated heparin flushes are recommended by the current guidelines. However,heparin increases clotting times and may result in fatal hemorrhages when doses are too high [50]. Frequent heparin flushing also affects the result of coagulation tests and disturbs the monitoring of heparin dosage [51]. Therefore, this consensus recommends avoiding heparin flush during vascular access placement for severe COVID-19 patients with coagulation dysfunction.

### Recommendation 17: For severe COVID-19 patients with coagulation dysfunction, the infusion dose of crystalloids and synthetic colloid should be controlled while maintaining adequate tissue perfusion

Previous studies have shown that Sonoclot was used to detect the coagulation status of healthy people after an input of 1 L of normal saline or succinyl gelatin. Normal saline can cause an average CR increase of 6% and an average ACT shortening of 15%, with an overall tendency towards hypercoagulation. However, succinyl gelatin can lead to an average CR reduction of 12% and an average peak time extension of 96%, with an overall tendency towards hypocoagulation [52]. As the fluid volume increases, coagulation dysfunction is further exacerbated because of dilution effect [53]. Additionally, isovolumetric dilution results in a significant decrease in platelet aggregation [54]. Therefore, severe COVID-19 patients with coagulation dysfunction should have strict controls over the excessive infusion of fluids, especially synthetic colloids [55].

### Recommendation 18: Influencing factors of coagulation dysfunction need to be actively controlled for severe COVID-19 patients during extracorporeal life support

Extracorporeal life support (ECLS) refers to the use of equipment in vitro to partially or completely replace organ functions to provide life support in case of life-threatening organ dysfunction. In a broad sense, ECLS includes ECMO that supports cardiopulmonary functions, RRT that supports renal function and ALSS that supports liver function [56].

During ECLS, the continuous contact between blood and external artificial surface materials leads to the activation of the coagulation system. The balance between coagulation and anticoagulation are affected, resulting in additional depletion of platelets and coagulation factors [57]. Anticoagulant drugs that prevent coagulation in the circuit may also affect the patient’s coagulation state, increasing the risk of bleeding-induced thrombocytopenia or inducing HIT [58]. Guru et al. [59] reported that thrombocytopenia occurred in 65% of critically ill patients who planned to receive CRRT, and thrombocytopenia occurred in another 20% of patients during CRRT. Patients with CRRT using heparin anticoagulation have more significant platelet decline than patients without CRRT using heparin anticoagulation. The 4 T’s scores indicate that most patients meet the diagnostic criteria for HIT, yet their positive antibody rate is not significantly high [60]. Choi et al. [61] reported that the rate of thrombocytopenia in patients receiving ECMO is as high as 83%. VA-ECMO is more likely than VV-ECMO to cause thrombocytopenia, because of platelet consumption caused by membrane oxygenator induced vWF aggregation [62]. The incidence of HIT can reach 20% during ECMO. Most patients show positive PF4 antibodies when treated with ECMO. The diagnosis of HIT needs to be considered if the ECMO circuit is frequently abnormal, the platelets progressively decrease and there are high levels of PF4 specific IgG antibodies [63].

Therefore, during ECLS, it is necessary to comprehensively assess the coagulation function, properly select anticoagulation strategies, positively control the factors of coagulation dysfunction. The specific targets are as follows: platelet count above 100 × 10^9^/L; prolonged PT less than 5 s; fibrinogen above 2 g/L; ATIII activity above 80% [30]. If ATIII cannot be monitored and a larger dose of unfractionated heparin is given to achieve anticoagulation effect, ATIII deficiency should be considered and fresh frozen plasma can be given. If significant coagulation dysfunction or active bleeding occurs during ECLS, the anticoagulation therapy can be stopped when necessary [64].

## Data Availability

Not applicable.

## Abbreviations

ACE: Angiotensin converting enzyme
ALSS: Artificial liver support system
ACT: Activated clotting time
APTT: Activated partial thromboplastin time
CDSS: Chinese DIC scoring system
DD: D-dimer
DIC: Disseminated intravascular coagulation
ECLS: Extracorporeal life support
FDP: Fibrin degradation products
HIT: Heparin-induced thrombocytopenia
ISTH: International Society on Thrombosis and Haemostasis
LMWH: Low molecular weight heparin
PT: Prothrombin time
SARS: Severe acute respiratory syndrome
TEG: Thromboelastograph
TT: Thrombin time

## References

[1] Zhu N, Zhang D, Wang W, Li X, Yang B, Song J (2020). A novel coronavirus from patients with pneumonia in China, 2019. N Engl J Med.

[2] Walls AC, Park YJ, Tortorici MA, Wall A, McGuire AT, Veesler D (2020). Structure, Function, and Antigenicity of the SARS-CoV-2 Spike Glycoprotein. Cell.

[3] Wu Z, McGoogan JM. Characteristics of and important lessons from the coronavirus disease 2019 (COVID-19) outbreak in China: summary of a report of 72 314 cases from the Chinese center for disease control and prevention. JAMA. 2020. 10.1001/jama.2020.2648. 10.1001/jama.2020.2648 32091533

[4] Wu C, Chen X, Cai Y, Xia J, Zhou X, Xu S, et al. Risk factors associated with acute respiratory distress syndrome and death in patients with coronavirus disease 2019 pneumonia in Wuhan, China. JAMA Intern Med. 2020. 10.1001/jamainternmed.2020.0994. 10.1001/jamainternmed.2020.0994 PMC7070509 32167524

[5] Tang N, Li D, Wang X, Sun Z. Abnormal Coagulation parameters are associated with poor prognosis in patients with novel coronavirus pneumonia. J Thromb Haemost. 2020. 10.1111/jth.14768. 10.1111/jth.14768 PMC7166509 32073213

[6] Kannan S, Shaik Syed Ali P, Sheeza A, Hemalatha K (2020). COVID-19 (Novel Coronavirus 2019) - recent trends. Eur Rev Med Pharmacol Sci.

[7] Chen Y, Guo Y, Pan Y, Zhao ZJ. Structure analysis of the receptor binding of 2019-nCoV. BiochemBiophys Res Commun. 2020. 10.1016/j.bbrc.2020.02.071. 10.1016/j.bbrc.2020.02.071 PMC7092824 32081428

[8] Wrapp D, Wang N, Corbett KS, Goldsmith JA, Hsieh CL, Abiona O (2020). Cryo-EM structure of the 2019-nCoV spike in the prefusion conformation. Science..

[9] Tian S, Hu W, Niu L, Liu H, Xu H, Xiao SY. Pulmonary pathology of early phase 2019 novel coronavirus (COVID-19) pneumonia in two patients with lung cancer. J Thorac Oncol. 2020. 10.1016/j.jtho.2020.02.010. 10.1016/j.jtho.2020.02.010 PMC7128866 32114094

[10] Chousterman BG, Swirski FK, Weber GF (2017). Cytokine storm and sepsis disease pathogenesis. Semin Immunopathol.

[11] Ding YQ, Bian XW (2020). Analysis of coronavirus disease-19 (covid-19). Chin J Pathol.

[12] Gralinski LE, Bankhead A, Jeng S, Menachery VD, Proll S, Belisle SE (2013). Mechanisms of severe acute respiratory syndrome coronavirus-induced acute lung injury. mBio.

[13] Novel Coronavirus Pneumonia Emergency Response Epidemiology Team (2020). The epidemiological characteristics of an outbreak of 2019 novel coronavirus diseases (COVID-19) in China. Chin J Epidemiol.

[14] Chinese Research Hospital Association of Critical Care Medicine, Youth Committee of Chinese Research Hospital Association of Critical Care Medicine (2020). Chinese experts consensus on diagnosis and treatment of severe and critical new coronavirus pneumonia. Chin Crit Care Med.

[15] Chen N, Zhou M, Dong X, Qu J, Gong F, Han Y (2020). Epidemiological and clinical characteristics of 99 cases of 2019 novel coronavirus pneumonia in Wuhan, China: a descriptive study. Lancet.

[16] Wang D, Hu B, Hu C, Zhu F, Liu X, Zhang J, et al. Clinical characteristics of 138 hospitalized patients with 2019 novel coronavirus-infected pneumonia in Wuhan, China. JAMA. 2020. 10.1001/jama.2020.1585. 10.1001/jama.2020.1585 PMC7042881 32031570

[17] Taylor FB, Toh CH, Hoots WK, Wada H, Levi M (2001). Scientific subcommittee on disseminated intravascular coagulation (DIC) of the international society on thrombosis and Haemostasis (ISTH). Scientific subcommittee on disseminated intravascular coagulation (DIC) of the international society on thrombosis and Haemostasis (ISTH). Towards definition, clinical and laboratory criteria, and a scoring system for disseminated intravascular coagulation. Thromb Haemost.

[18] Thrombosis and Hemostasis Group, Hematology Society of Chinese Medical Association (2017). Consensus of Chinese experts on diagnosis of disseminated intravascular coagulation (version 2017). Chin J Hematol.

[19] Wu Y, Luo L, Niu T, Han Y, Feng Y, Ding Q (2017). Evaluation of the new Chinese disseminated intravascular coagulation scoring system in critically ill patients: a multicenter prospective study. Sci Rep.

[20] Hartmann J, Murphy M, Dias JD (2020). Viscoelastic hemostatic assays: Moving from the laboratory to the site of care-A review of established and emerging technologies. Diagnostics (Basel).

[21] Liu HQ, Zeng QB, Song JC, Yang Y, Zhong LC, Yu T (2019). A comparative study of thromboelastography and centuryclot coagulation analyzer in monitoring coagulation function in intensive care patients. Clin J Med Off.

[22] Wesley B, Matthew L, Michael M, Alexandra E, Christopher S, Richard J, et al. The ability of thromboelastography to detect hypercoagulability: a systematic review & meta-analysis. J Orthop Trauma. 2019. 10.1097/BOT.0000000000001714.

[23] Emergency Medicine Branch of Chinese Medical Education Association, Cardio Cerebrovascular Group of Emergency Medicine Branch of Chinese Medical Association, Emergency Expert Consensus Group of Acute Thrombotic Diseases (2019). Chinese consensus on antithrombotic treatment of acute thrombotic diseases. Chin Emerg Med.

[24] Expert Group on Novel Coronavirus Disease Clinical Treatment in Shanghai (2020). Expert consensus on comprehensive treatment of 2019 coronavirus in Shanghai. Chin J Infect dis.

[25] Group of Thrombosis and Hemostasis, Hematology Branch, Chinese Medical Association (2012). Chinese expert consensus on diagnosis and treatment of disseminated intravascular coagulation (2012). Chin J Hematol.

[26] Cvirn G, Tafeit E, Hoerl G, Janschitz M, Wagner T, Juergens G (2010). Heparinase-modified thrombelastometry: inactivation of heparin in plasma samples. Clin Lab.

[27] Deng XP, Zeng QB, Hu W, Lin QW, Chen T, Song JC (2018). Application of heparinase-modified thromboelastography monitoring heparin during continuous blood purification. J Sou Chin Nati l Defe Med Sci.

[28] Yang XH, Sun RH, Zhao MY, Chen EZ, Liu J, Wang HL (2020). Novel coronavirus pneumonia treatment for severe new type coronavirus pneumonia expert advice. Chin Med J.

[29] Committee of Extracorporeal Life Support of Chinese Medical Association (2018). Consensus of experts on extracorporeal membrane oxygenation in adults. Chin Med J.

[30] Committee of Critical Care Medicine, Chinese Association of Chest Physician, Chinese Medical Doctor Association, Critical Care Medicine Group, Chinese Thoracic Society, Chinese Medical Association (2019). Recommendations for clinical application of extracorporeal membrane oxygenation in adults severe acute respiratory failure. Chin J Tuberc Respir Dis.

[31] Warkentin TE (2016). Clinical picture of heparin-induced thrombocytopenia (HIT) and its differentiation from non-HIT thrombocytopenia. ThrombHaemost.

[32] Warkentin TE, Greinacher A, Gruel Y, Aster RH, Chong BH (2011). Scientific and standardization Committee of the International Society on thrombosis and Haemostasis. Laboratory testing for heparin-induced thrombocytopenia: a conceptual framework and implications for diagnosis. J Thromb Haemost.

[33] Cuker A, Arepally GM, Chong BH, Cines DB, Greinacher A, Gruel Y (2018). American Society of Hematology 2018 guidelines for management of venous thromboembolism: heparin-induced thrombocytopenia. Blood Adv.

[34] Squizzato A, Hunt BJ, Kinasewitz GT, Wada H, Ten Cate H, Thachil J (2016). Supportive management strategies for disseminated intravascular coagulation. An international consensus. Thromb Haemost.

[35] Waters JH (2014). Role of the massive transfusion protocol in the management of haemorrhagic shock. Br J Anaesth.

[36] Peng HT, Nascimento B, Beckett A (2018). Thromboelastography and thromboelastometry in assessment of fibrinogen deficiency and prediction for transfusion requirement: a descriptive review. Biomed Res Int.

[37] National Health Commission of the PRC (2018). Transfusion of whole blood and blood components: WS / T 623–2018 [S].

[38] Newland A, Bentley R, Jakubowska A, Liebman H, Lorens J, Peck-Radosavljevic M (2019). A systematic literature review on the use of platelet transfusions in patients with thrombocytopenia. Hematology.

[39] Stettler GR, Sumislawski JJ, Moore EE, Nunns GR, Kornblith LZ, Conroy AS (2018). Citrated kaolin thrombelastography (TEG) thresholds for goal-directed therapy in injured patients receiving massive transfusion. J Trauma Acute Care Surg.

[40] Zeng QB, Song JC, Hu W, Shang F, Lin QW, Zhong LC (2019). Clinical study of thromboelastography in the indication of platelet transfusion in severe trauma. Chin J Transfu.

[41] Zhang ZL, Chen YP, Tao CH, Liu XH, Li MY, Zhou X (2016). Establishment of reference intervals and transfusion criterion for Sonoclot analysis. J Huazhong Univ Sci Technolog Med Sci.

[42] Hansson EC, Shams Hakimi C, Astrom-Olsson K, Hesse C, Wallén H, Dellborg M (2014). Effects of ex vivo platelet supplementation on platelet aggregability in blood samples from patients treated with acetylsalicylic acid, clopidogrel, or ticagrelor. Br J Anaesth.

[43] Holme PA, Tjønnfjord GE, Batorova A (2018). Continuous infusion of coagulation factor concentrates during intensive treatment. Haemophilia..

[44] Liver Failure and Artificial Hepatology Group, Infectious Disease Society of Chinese Medical Association, Liver Disease Society of Chinese Medical Association, Severe Liver Disease and Artificial Hepatology Group (2019). Guidelines for diagnosis and treatment of liver failure (2018 edition). Chin J Infect Dis.

[45] Schwartz J, Padmanabhan A, Aqui N, Balogun RA, Connelly-Smith L, Delaney M (2016). Guidelines on the use of therapeutic apheresis in clinical pactice-evidence-based approach from the writing committee of the American Society for Apheresis: The seventh special issue. J Clin Apher.

[46] Putzu A, Schorer R, Lopez-Delgado JC, Cassina T, Landoni G (2019). Blood purification and mortality in sepsis and septic shock: a systematic review and meta-analysis of randomized trials. Anesthesiology.

[47] Hadem J, Hafer C, Schneider AS, Wiesner O, Beutel G, Fuehner T (2014). Therapeutic plasma exchange as rescue therapy in severe sepsis and septic shock: retrospective observational single-center study of 23 patients. BMC Anesthesiol.

[48] National Infectious Diseases Clinical Medicine Center, National Laboratory of Infectious Diseases Diagnosis and Treatment (2020). Expert consensus on novel coronavirus pneumonia treated with artificial liver blood purification system. Chin J Clin Infect Dis.

[49] Nanchal R, Subramanian R, Karvellas CJ, Hollenberg SM, Peppard WJ, Singbartl K (2020). Guidelines for the management of adult acute and acute-on-chronic liver failure in the ICU: cardiovascular, endocrine, hematologic, pulmonary, and renal considerations. Crit Care Med.

[50] Peterson K (2013). The development of central venous access device flushing guidelines utilizing an evidence-based practice process. Pediatr Nurs.

[51] Alizadehasl A, Ziyaeifard M, Peighambari M, Azarfarin R, Golbargian G, Bakhshandeh H (2015). Avoiding heparinization of arterial line and maintaining acceptable arterial waveform after cardiac surgery: a randomized clinical trial. Res Cardiovasc Med.

[52] Coats TJ, Brazil E, Heron M, MacCallum PK (2006). Impairment of coagulation by commonly used resuscitation fluids in human volunteers. Emerg Med J.

[53] Orfeo T, Gissel M, Haynes LM, Pusateri A, Mann KG, Brummel-Ziedins KE (2018). Hemodilution and endothelial cell regulation of whole blood coagulation. Mil Med.

[54] Scott KJ, Shteamer JW, Szlam F, Sniecinski RM (2019). Platelet function, but not thrombin generation, is impaired in acute normovolemic hemodilution (ANH) blood. J Clin Anesth.

[55] Liebrecht LK, Newton J, Martin EJ, Wickramaratne N, Jayaraman S, Han J (2018). Thromboelastographic analysis of novel polyethylene glycol based low volume resuscitation solutions. PLoS One.

[56] Ronco C, Ricci Z, Husain-Syed F (2019). From multiple organ support therapy to extracorporeal organ support in critically ill patients. Blood Purif.

[57] Oudemans-van Straaten HM (2015). Hemostasis and thrombosis in continuous renal replacement treatment. Semin Thromb Hemost.

[58] Akhoundi A, Singh B, Vela M, Chaudhary S, Monaghan M, Wilson GA (2015). Incidence of adverse events during continuous renal replacement therapy. Blood Purif.

[59] Guru PK, Singh TD, Akhoundi A, Kashani KB (2016). Association of thrombocytopenia and mortality in critically ill patients on continuous renal replacement therapy. Nephron.

[60] Holmes CE, Huang JC, Cartelli C, Howard A, Rimmer J, Cushman M (2009). The clinical diagnosis of heparin-induced thrombocytopenia in patients receiving continuous renal replacement therapy. J Thromb Thrombolysis.

[61] Choi JH, Luc JGY, Weber MP, Reddy HG, Maynes EJ, Deb AK (2019). Heparin-induced thrombocytopenia during extracorporeal life support: incidence, management and outcomes. Ann Cardiothorac Surg.

[62] Steiger T, Foltan M, Philipp A, Mueller T, Gruber M, Bredthauer A (2019). Accumulations of von Willebrand factor within ECMO oxygenators: potential indicator of coagulation abnormalities in critically ill patients?. Artif Organs.

[63] Vayne C, May MA, Bourguignon T, Lemoine E, Guery EA, Rollin J (2019). Frequency and clinical impact of platelet factor 4-specific antibodies in patients undergoing extracorporeal membrane oxygenation. Thromb Haemost.

[64] Hermann A, Schellongowski P, Bojic A, Robak O, Buchtele N, Staudinger T (2019). ECMO without anticoagulation in patients with disease-related severe thrombocytopenia: feasible but futile?. Artif Organs.

